# Safety study of Rift Valley Fever human vaccine candidate (DDVax) in mosquitoes

**DOI:** 10.1111/tbed.14415

**Published:** 2022-01-05

**Authors:** Corey L. Campbell, Trey K. Snell, Susi Bennett, John H. Wyckoff, Darragh Heaslip, Jordan Flatt, Emma K. Harris, Daniel A. Hartman, Elena Lian, Brian H. Bird, Mark D. Stenglein, Richard A. Bowen, Rebekah C. Kading

**Affiliations:** ^1^ Department of Microbiology Immunology, and Pathology Center for Vector‐Borne Infectious Diseases Colorado State University Fort Collins Colorado; ^2^ BioMARC, Infectious Diseases Research Center, Colorado State University Fort Collins Colorado; ^3^ School of Veterinary Medicine One Health Institute University of California Davis California

**Keywords:** *Aedes*, arbovirus, *Culex*, vaccine, vector competence, vector‐borne disease

## Abstract

Rift Valley fever virus (RVFV) is a mosquito‐borne pathogen with significant human and veterinary health consequences that periodically emerges in epizootics. RVFV causes fetal loss and death in ruminants and in humans can lead to liver and renal disease, delayed‐onset encephalitis, retinitis, and in some cases severe haemorrhagic fever. A live attenuated vaccine candidate (DDVax), was developed by the deletion of the virulence factors NSs and NSm from a clinical isolate, ZH501, and has proven safe and immunogenic in rodents, pregnant sheep and non‐human primates. Deletion of NSm also severely restricted mosquito midgut infection and inhibited vector‐borne transmission. To demonstrate environmental safety, this study investigated the replication, dissemination and transmission efficiency of DDVax in mosquitoes following oral exposure compared to RVFV strains MP‐12 and ZH501. Infection and dissemination profiles were also measured in mosquitoes 7 days after they fed on goats inoculated with DDvax or MP‐12. We hypothesized that DDVax would infect mosquitoes at significantly lower rates than other RVFV strains and, due to lack of NSm, be transmission incompetent. Exposure of *Ae. aegypti* and *Cx. tarsalis* to 8 log_10_ plaque forming units (PFU)/ml DDVax by artificial bloodmeal resulted in significantly reduced DDVax infection rates in mosquito bodies compared to controls. Plaque assays indicated negligible transmission of infectious DDVax in *Cx. tarsalis* saliva (1/140 sampled) and none in *Ae. aegypti* saliva (0/120). Serum from goats inoculated with DDVax or MP‐12 did not harbour detectable infectious virus by plaque assay at 1, 2 or 3 days post‐inoculation. Infectious virus was, however, recovered from *Aedes* and *Culex* bodies that fed on goats vaccinated with MP‐12 (13.8% and 4.6%, respectively), but strikingly, DDvax‐positive mosquito bodies were greatly reduced (4%, and 0%, respectively). Furthermore, DDVax did not disseminate to legs/wings in any of the goat‐fed mosquitoes. Collectively, these results are consistent with a beneficial environmental safety profile.

## 1 INTRODUCTION

Rift Valley fever phlebovirus (RVFV) (family *Phenuiviridae*, genus *Phlebovirus*) is a mosquito‐borne virus that causes periodic epizootic outbreaks across Africa and the Arabian peninsula (Al‐Afaleq & Hussein, 2011; Nguku et al., 2010). In ruminants, primarily sheep, goats, camels and other camelids, Rift Valley fever often manifests in near universal foetal death at all stages of gestation (Coetzer, 1982; Odendaal et al., 2020) with significant adult animal deaths often due to acute virus induced hepatic and renal pathology (Odendaal et al., 2019; Odendaal et al., 2021; Wichgers Schreur et al., 2021). Though most human cases are typically self‐limiting with mild to moderate symptoms (Al‐Hazmi et al., 2003; McElroy et al., 2018), kidney and/or eye damage, severe anaemia, haemorrhagic fever and miscarriage can occur (Baudin et al., 2016; Coetzer, 1982; Madani et al., 2003; Oymans et al., 2020).

Over 40 species of mosquitoes, primarily *Culex* and *Aedes* species, are competent vectors for RVFV (reviewed in Lumley et al., 2017), and some are present on multiple continents (Lumley et al., 2018). Mosquitoes are able to imbibe RVFV from animals with relatively low viral titres (Turell et al., 2008; Wichgers Schreur et al., 2021). Following periods of heavy rainfall, which stimulate rapid increases in vector mosquito populations, RVFV re‐emerges periodically in explosive epizootics (Al‐Afaleq & Hussein, 2011; Nguku et al., 2010). Of note, the specific composition of infected mosquito species varies depending on the region (Sang et al., 2010), consistent with the contribution of multiple species to a given outbreak. In the absence of humans and livestock, RVFV cycles between mosquitoes and wild ruminants (Britch et al., 2013; Clark et al., 2018). Between epizootics, RVFV is maintained at low levels in livestock (Lichoti et al., 2014).

Due to the potential for RVFV to cause a public health emergency, in 2018 the World Health Organization listed this virus as a research and development blueprint priority pathogen (Mehand et al., 2018). A number of vaccine candidates have been developed, including formalin inactivated (Pittman et al., 1999; Randall et al., 1962) and live attenuated strains (Faburay et al., 2017; Ikegami et al., 2015; Smithburn, 1949). However, teratogenic effects in animals (Anthony et al., 2021; Hunter et al., 2002; Makoschey et al., 2016) or the need for boosters to maintain protective immunity (Bird et al., 2009; Botros et al., 2006) presented challenges for further development of these early candidates. For example, the live‐attenuated vaccine strain, MP‐12, was developed through 5‐fluorouracil mutagenesis of the parental strain ZH548 (Caplen et al., 1985). MP‐12 is efficacious in protecting sheep (Miller et al., 2015; Morrill et al., 2013); however, it showed low levels of abortogenesis and teratogenic effects (Hunter et al., 2002) and also showed transmission potential in mosquitoes (Turell & Rossi, 1991). More recent developmental strategies have exploited reverse engineering to produce segmented (Wichgers Schreur et al., 2020) or gene deletion vaccine candidates (Bird et al., 2008; Bird et al., 2011).

Availability of a safe and effective human vaccine against RVFV is essential to protect the health of people in endemic regions and a preparatory measure for the anticipated cross‐border spread and establishment in new geographic areas. In summary, to date, there is currently no commercially available and fully FDA‐approved RVFV human vaccine. To meet this critical health need, a human vaccine candidate (DDVax), a double deletion construct of the parental wild‐type strain ZH501, was generated using a reverse genetics approach wherein both the NSs (non‐structural, S segment) and NSm (non‐structural, M segment) virulence genes were removed (Bird et al., 2008). NSs is expressed from the viral S segment (Ikegami et al., 2009) and is a multi‐functional protein that antagonizes host cell interferon responses (Le May et al., 2008). The viral M segment encodes two major glycoproteins and multiple open reading frames in the NSm coding regions, which is required for efficient dissemination in mosquitoes (Crabtree et al., 2012). Neither NSs nor NSm are required for viral replication in interferon‐deficient cell culture, and the attenuated DDVax vaccine candidate was shown to be safe and immunogenic in a variety of animal species with the added benefit of inhibited replication and transmission in mosquitoes (Bird et al., 2008; Bird et al., 2011; Crabtree et al., 2012; Kading, Crabtree, et al., 2014; Smith et al., 2018). More specifically, vaccination with the single deletion NSs strain in non‐human primates showed reasonable protection against viral challenge (Smith et al., 2018).

The objective of this study was to confirm that DDVax produced under Good Manufacturing Practices behaved as previously described and exhibited a highly favourable environmental safety profile, specifically in the lack of transmission in potential mosquito vectors. Here, we describe characterization of RVFV DDVax in mosquitoes in two experimental phases: (1) mosquito oral challenges via artificial feeding and (2) mosquito feeding on DDVax inoculated goats. Features of vector competence were measured in two competent mosquito species, *Culex tarsalis* Coquillett and *Aedes aegypti* Linneaus, to determine infection, dissemination and transmission potential, using reverse transcriptase‐quantitative PCR (RT‐qPCR) and infectious virus plaque assay. Vertebrate‐to‐vector transmission from DDVax‐inoculated goats to mosquitoes was also measured. Collectively, these experiments provided an important comparison of vector competence of mosquitoes exposed to DDVax (Bird et al., 2008), ZH501, the parental wild‐type virus and MP‐12, an existing vaccine virus strain (Turell & Rossi, 1991).

## 2 METHODS

### 2.1 Generation of DDVax pilot material

Synthesized RVFV genomic segments (S, M and L) containing the DDVax specific deletions of the NSs and NSm genes were inserted into three separate DNA plasmids. Details of the deletion of NSs and NSm have been described in Bird et al. (2008), Bird et al. (2007) and Gerrard et al. (2007). The three DDVax plasmids and with a fourth plasmid encoding the T7 RNA polymerase were mixed in 0.625 μg quantities with the transfection reagent LT‐1 (Mirus) at a ratio of 6:1 and transferred onto monolayers (confluency ∼80 to 85%) of BKH‐21 (ATCC) cells propagated in Eagle's essential minimal media (ATCC) supplemented with 10% FBS (Atlas Biologicals). Three days post‐transfection, the cell supernatant was clarified by low‐speed centrifugation and passaged four times on confluent monolayers of serum‐free Vero cells (Vivaldi Biosciences; derived from a Master Cell Bank stock which had been previously tested for vaccine manufacturing compliance) generating a research virus stock (RVS) of DDVax material. Serum‐free Vero cells (ATCC CCL‐81, 2 × 10^4^ cells/cm^2^) were grown in OptiPro Serum‐Free Media (SFM; ThermoFisher) with 4 mM GlutaMAX™ (ThermoFisher) at 37°C and 5% CO_2_. Cells were expanded into 3 × 10 layer 6360 cm^2^ CellSTACK^®^ (Corning). For each passage, cells were seeded at either 2.0 × 10^4^ cells/cm^2^ for 48 ± 8 h or 1.5 × 10^4^ cell/cm^2^ for 72 ± 8 h. Cell harvest was performed using TrypLE Select (LifeTech). Cells were centrifuged at 500 × *g* for 5 min at 18°C and resuspended in OptiPro SFM. Cell enumeration was performed using a Vi‐Cell Cell Viability Analyzer.

DDVax material was prepared by passaging the RVS material on serum‐free Vero cell monolayers of serum‐free cells in 3 × 10 layer or 1 × 1 layer CellSTACK^®^. Vero cells were infected with the DDVax Research Virus Stock (RVS), Lot # N16‐5‐20‐RV at a multiplicity of infection (MOI) of 0.0005 PFU/cell. Infection volume used for each 10 layer CellSTACK^®^ was 1300 ml and the infection volume for the 1 layer CellSTACK^®^ was 130 ml. Infected cultures were then incubated at 37°C and 5% CO_2_ for 3 days, at which time the flasks were examined for cytopathic effects (CPE). Virus was harvested when 70% CPE was attained. The cell culture supernatant was treated with 50,000 U/L benzonase (EMD Millipore), 1.5 mM MgCl_2_, and incubated for 60 min at 37°C and 5% CO_2_, with rocking at 10‐min intervals. The benzonase‐treated pool was clarified by centrifugation at 3000 × g for 15 min at 18°C and concentrated sixfold by ultrafiltration (UF) over a 500 kD Hollow Fiber Membrane (Repligen). The concentrated UF pool was diafiltered into a buffer containing 0.2 M NaCl, 10 mM sodium phosphate, 4% sucrose, 5 mM GlutaMAX™, pH 7.4 ± 0.1. Buffer exchange was performed for 10 diavolumes. Diafiltration was performed at a target shear rate of 3000 s^−1^ and TMP setpoint of 5 psi. The pool underwent a final polishing step to remove residual host cell proteins, HCD, benzonase and endotoxin by size exclusion chromatography using a 70 ml Capto Core 700 column (Cytiva). The UF/DF pool was passed through the column and then was washed with 100 ml of 0.2 M NaCl, 10 mM sodium phosphate, 4% sucrose, 5 mM glutamax, pH 7.4 ± 0.1. The pool was then filtered using a Supor EKV 0.2 μm filter (Pall) and divided into 0.5, 1 and 50 ml aliquots and stored at ≤ −60°C.

### 2.2 DDVax sequencing and analysis

DDVax RNA was prepared from viral passages 1 through 5 using Trizol reagent (ThermoFisher) as previously described (Hoon‐Hanks et al., 2018). Independent passage 5 preparations were used to generate pilot stock used in the mosquito experiments, as described below. Illumina shotgun sequencing libraries were prepared from total RNA using the Kapa RNA HyperPrep kit following the manufacturer's protocol. Dual indexed libraries were sequenced on an Illumina NextSeq 500 sequencer to generate single‐end 150 nt reads.

We used two complementary approaches to detect and quantify viral variants. First, we used the lofreq tool to identify single nucleotide variants (SNVs) and short insertions and deletions (Wilm et al., 2012). Second, we used DI‐tector to identify structural variants including longer deletions and insertions and copy back defective viral genomes (DVGs) (Beauclair et al., 2018; Vignuzzi & Lopez, 2019). These tools were run as part of a reproducible Nextflow pipeline, available at https://github.com/stenglein‐lab/viral_variant_caller/releases/tag/DDVax_paper_release. Software dependencies and reference sequences (DDVax) are captured in this version‐controlled release and in the conda environment contained therein.

To quantify variants, adapter‐derived and low‐quality bases were trimmed using Cutadapt (Martin, 2011). Host cell‐derived reads were removed using bowtie2 to align reads to the *Chlorocebus sabeus* genome, accession GCF_000409795.2 (Langmead & Salzberg, 2012). Host‐ and quality‐filtered reads were aligned to the S, M and L segment RVFV/DDVax reference sequences using the BWA aligner (Langmead & Salzberg, 2012; Li & Durbin, 2009). The reference sequences consisted of the RVFV‐derived portions of the DDVax plasmid sequences. To improve accuracy of structural variant (indel) calls, base quality scores were recalibrated using GATK (McKenna et al., 2010). SNVs and structural variants were called using LoFrEquation (Wilm et al., 2012). The minimum depth of coverage to call a variant was set at 40× coverage. SnpEff and SnpSift were used to predict the functional impact of variants (Cingolani, Patel, et al., 2012; Cingolani, Platts, et al., 2012). DVGs were identified using the DI‐tector tool (Beauclair et al., 2018). Outputs of these analyses were tabulated, processed and visualized in R using tidyverse packages, with scripts available at the GitHub repository linked above (Wickham et al., 2018). Variants with frequencies ≥3% were reported (Grubaugh et al., 2019).

### 2.3 Virus strains

Stocks of DDVax were produced as described above. The DDVax preparations used for the mosquito infections and goat vaccinations were pilot material, each representing independent passage five from the research stock virus. The filtered pool from pilot #1 was used for mosquito vector competence, and pilot #2 was used for the goat vaccinations. A high passage strain of MP‐12 (passage unknown, local lab passage 3), which was a gift from the US Department of Agriculture, was also used. ZH501 strain virus was obtained from R. Bowen. V1 (Vero) passage stock was passaged twice in Vero cells to obtain V3 stocks used for this study.

### 2.4 Mosquitoes

The Poza Rica *Ae. aegypti* strain was colonized in 2012 from collections in the state of Veracruz, Mexico (Vera‐Maloof et al., 2015). The *Cx. tarsalis* Kern National Wildlife Refuge (KNWR) colony (Oviedo et al., 2011), established in 1952, was obtained from the Centers for Disease Control and Prevention (Fort Collins, CO). Mosquito colonies were maintained at 24–26°C (*Culex*) or 28°C (*Aedes*) on a 12:12 light:dark cycle; adults were fed water and sucrose ad libitum. Larvae were reared on TetraMin fish food (http://www.tetra‐fish.com/) that had been ground in a coffee grinder.

### 2.5 Vector competence

All virus growth and mosquito experiments were performed in standard biosafety level 3 (BSL‐3) level containment. All ZH501 feedings and mosquito incubation steps were performed in the animal BSL‐3 laboratory spaces registered for work with this select agent, and in compliance with select agent regulations and CSU biosafety protocol 19–073B.

Adult mosquitoes (4–10 days old) were provided an oral, artificial meal containing freshly grown RVFV. To approximate titres of 7 log_10_ PFU/ml, frozen stocks of DDVax, MP‐12 or ZH501 RVFV were used to infect foetal bovine serum (FBS)‐dependent Vero cells (ATCC CCL‐81), each at an MOI of 0.01. This was because frozen stock virus was previously determined to not be infectious to mosquitoes. At 3 dpi, viral supernatant was mixed 1:1 in defibrinated calf blood, with the addition of 1 mM ATP and 0.075% sodium bicarbonate. Mosquitoes were fed for 1 to 1 ½ h using either a water‐jacketed feeder (https://lillieglassblowers.com) for DDVax and MP‐12 or a haemotek (http://hemotek.co.uk/), in the case of ZH501. Fully engorged mosquitoes were separated into cartons and provided sucrose and water ad libitum. Mosquitoes were held for 14 days at ∼80% humidity and 28°C. Infectious blood meal titres were determined through back titration.

At 14 days post‐challenge, mosquitoes were anesthetized at 4°C, then held on ice during processing. Tissue samples were dissected, then placed in separate tubes of 250 μl mosquito diluent (DMEM, 20% heat‐inactivated FBS, 50 μg/ml Pen‐Strep, 50 μg/ml gentamicin and 2.5 μg/ml amphotericin B), as follows: legs and wings were removed for determination of viral dissemination. Saliva was collected for determination of transmission potential. The mosquito proboscis was placed in a capillary tube containing type B immersion oil (Bioworld, SKU‐ 21750002) and allowed to salivate for 30–60 min. At that time, the capillary tube was removed and placed in a tube containing 250 μl mosquito diluent and centrifuged at 14,000 × *g* for 3 min. Lastly, each remaining body was also placed in a separate tube with 250 μl diluent, for measurement of infection status. Samples were homogenized on a Qiagen Tissuelyzer (Qiagen) at 30 beats/s frequency for 30 s, then pelleted at 14,000 × *g* in a centrifuge (Eppendorf) at 4°C for 3 min. Samples were stored in −80°C.

### 2.6 RNA extractions

RNA was extracted from saliva, legs/wings and bodies samples generated from in vitro vector competence experiments in preparation of RT‐qPCR. RNA extractions of individual 50 μl sample aliquots were performed using the Applied Biosystems MagMax‐96 Viral RNA extraction kit (AMB1836‐5, Thermofisher) following the manufacturer's protocol for manual extraction methods (MAN0017826). Linear polyacrylamide was used as a carrier in place of carrier RNA. Extractions were eluted into 50 μl elution buffer and stored in 96‐well plates at −80°C.

### 2.7 Reverse transcription quantitative PCR (RT‐qPCR)

RNA copy number standards were developed by amplifying a portion of the L segment from 20 ng plasmid bearing the full‐length gene (Bird et al., 2008). The RVFL2173_T7_F amplification forward primer contained a T7 promoter; RVFL3542_R was the reverse primer (Table S1). 100 ng input of PCR product was used in vitro transcription reactions that incubated for 5 h at 37°C using the manufacturer's recommendations. Transcription products were stored in 5 μl aliquots at −80°C; they were quantitated using a Qubit fluorometer (ThermoFisher) using the manufacturer's recommendations. For RT‐qPCR, fresh aliquots of in vitro transcription reactions were serially diluted in 10‐fold increments to generate standard curves to relate copy number to raw cycle threshold (Ct value). One standard plate was run for all samples screened on a given day. A representative standard curve was *y* = −3.3111x + 36.655 *R* ^2 ^= .9976, where *y* = Ct value and *x* = log_10_ RNA copy number.

RT‐qPCR was performed in duplicate using 5 μl sample or RNA standards and run on a QuantStudio 2.0 qPCR platform (Applied Biosystems). Calculated virus amounts were adjusted to account for RNA copy number per tissue. The following primers were used to quantitate RVFV RNA in all samples: RVFL‐2912fwdgg, RVFL‐2971revAC and RVFL‐2950‐Probe (Table S1) (Bird et al., 2007). TaqMan Fast Virus 1‐Step Master Mix (Applied Biosystems) was used with final primer concentrations of 500 nM and a probe concentration of 100 nM. Samples and standards were loaded into 96‐well plates and run using fast cycling mode on an AB QuantStudio machine, using the manufacturer's recommended settings. The cycling conditions were as follows: 50°C, 5 min (1 cyc), 95°C, 20 s (1 cyc), 95°C, 3 s and 60°C, 30 s (40 cyc).

### 2.8 DDVax dose–response experiment

A dose–response experiment was performed as a follow‐up to the mosquito vector competence challenges, which were administered with only a single high titre of over 8.0 log_10_ PFU/ml. The purpose of this experiment was to test the hypothesis that *Cx. tarsalis* DDVax infection rates vary as a function of virus titre in the artificial blood meal. *Cx. tarsalis* were exposed to oral bloodmeals at 6.2, 4.5 or 3.5 log_10_ PFU/ml and held for 14 days at 28°C, rH 80%. At 14 days post‐feeding legs/wings, saliva and bodies were harvested into mosquito diluent as above in individual tubes and stored at −80°C. Sample processing was performed as described above.

### 2.9 Goat virus inoculations and mosquito challenge

Mature female, non‐pregnant dairy goats of multiple breeds were acquired from a commercial dairy and housed in an Animal Bio‐Safety Level 3 facility for the duration of the experiment. Goats were inoculated with 5.6 log_10_ PFU freshly grown MP‐12 or 6.6 log_10_ PFU DDVax, as determined by plaque assay. Blood was drawn from goat jugular vein at days 1, 2 and 3 post‐inoculation into gel serum separator tubes (Becton Dickson, https://www.bd.com/); serum was collected by spinning at 1200 × *g* for 10 min. Serum was aliquoted and stored at −80°C. Serum samples were titred by plaque assay, and RNA was extracted for detection and quantification of viral RNA.

For mosquito feeding, goats were manually restrained, and mosquitoes in cartons with mesh bottoms were held against patches of clipped fur for about 30 min to allow feeding on days 1 and 2 post‐inoculation (Figure S1). Because *Cx. tarsalis* mosquitoes did not feed well on goats, on day 3 post‐inoculation, *Cx. tarsalis* and *Ae. aegypti* were exposed in the laboratory to freshly collected goat blood (collected into EDTA tubes; Becton Dickson, https://www.bd.com/) using a water jacketed feeding apparatus heated to 37°C. Engorged mosquitoes were held for 7 days at 28°C, rH 80%. At 7 days post‐feeding, bodies and legs/wings were placed in individual tubes containing mosquito diluent (see above). Samples were homogenized on a Qiagen Tissuelyzer (Qiagen) at 30 beats/s frequency for 30 s, then pelleted at 14,000 × *g* in a centrifuge at 4°C for 3 min. Tubes were stored in −80°C. Infectious virus (CPE+/–) was measured by plaque assay using 100 μl undiluted sample in duplicate to determine the frequency of mosquito bodies bearing infectious DDVax virus or MP‐12 RVFV (control). For those with RVFV‐positive bodies, legs/wings were also titrated by plaque assay to determine the frequency of mosquitoes with disseminated infectious virus.

### 2.10 Virus titrations

Vero cells were grown to ≥95% confluency in Dulbecco's modified eagle media DMEM (5% foetal bovine serum (Atlas Biologicals), 1% sodium bicarbonate, 1% non‐essential amino acids, no phenol red) in 6‐ or 12‐well plates. Tenfold serial dilutions of virus stocks and blood meal aliquots in media were performed. Mosquito samples were used undiluted. In vitro challenged mosquito samples had already undergone one freeze‐thaw cycle prior to infectious virus detection. For each dilution or sample, 100 μl of sample was added to wells, then incubated with rocking for 1 h, followed by an overlay [0.4% agarose (Lonza Rockland) in DMEM]. At 2 days post‐infection, overlays [0.33% neutral red (Sigma N2889), 2% agarose in supplemented DMEM] were applied. Plates were read after 24 h. Ambiguous plaques were more closely examined under an inverted microscope at 40× magnification to better confirm CPE.

### 2.11 Insect cell culture virus growth curves

The insect cell lines used for this study were Ct cells (lab passage 2), derived from *Cx. tarsalis* embryos (Centers for Disease Control and Prevention) (Chao & Ball, 1976), Aag2 *Ae. aegypti* high passage cells (lab passage 2), also derived from embryos (Chao & Ball, 1976), and ATC‐10 (CCL‐125 (ATCC), lab passage 1), an *Ae. aegypti* larval‐derived cell line (Singh, 1971). Growth curves of ZH501, MP‐12 and DDVax were performed in mosquito cell culture (ATC‐10, Aag2, CT) using Schneider's media [10% FBS (or 20% FBS for ATC‐10s), 1% non‐essential amino acids, 1% L‐glutamine, 1% penicillin/streptomycin]. An MOI of 0.01 was used for all infections, and three biological replicates were performed for each growth series. Infected cells were held at 30°C, 60% rH and 5% CO_2_. Aliquots were removed at daily timepoints for 1–6 days post‐infection (dpi). At each timepoint, 400 μl cell culture supernatant was removed, and media was replaced. Aliquots of culture supernatant were supplemented with 20% FBS as a cryoprotectant and stored at −80°C until titrations were performed.

### 2.12 MP‐12 genotype confirmation

Previously characterized mutations (Ikegami et al., 2015), as well as the purity of the virus stock, were confirmed in MP‐12‐infected mosquitoes by Sanger sequencing using primers listed in Table S1. Specifically, 20 pooled RNA samples from individual *Culex* legs/wings of replicate #2 of the artificial bloodmeal experiment were subjected to one‐step RT‐PCR (Qiagen) according to the manufacturer's suggestions. Cycling parameters were 30 min at 50°C, 15 min at 95°C, then 35 cycles (94°C, 0.5 min, 55°C, 0.5 min, 72°C, 2 min), followed by 72°C for 10 min. PCR products were confirmed by gel electrophoresis; extraneous primers were removed using Exo‐SAP‐IT (Applied Biosystems), using the manufacturer's protocol. Samples were Sanger sequenced (Genewiz). Diagnostic MP‐12 point mutations were confirmed at G3750A, G4368T and A5208G (Ikegami et al., 2015) on the L segment (DQ375404.1). Point mutations at U795C, G857A, A3564G, A3621G, A3644G and A3660U on the M segment (DQ380208.1) were also confirmed.

### 2.13 Data analysis

Per cent infection was determined by calculating the proportion of viral RNA‐positive mosquito bodies for the combined total number of mosquito RNA samples. Dissemination was determined by calculating the proportion of legs/wings RNA samples with detectable RVFV RNA against the total number of mosquitoes exposed. Transmission was determined by calculating the proportion of saliva RNA samples that were RVFV‐RNA positive against the total number of mosquitoes exposed. Per cent of saliva expectorants containing infectious virus were also calculated by determining the proportion of saliva samples producing detectable CPE by plaque assay among the total number of individuals tested. The percentage of RVFV‐infected mosquitoes after feeding on inoculated goats was determined by calculating plaque‐positive mosquito bodies per total number of mosquitoes assayed. RVFV growth curve titres were analysed by calculating the highest dilution containing countable plaques and multiplying that by the dilution factor to obtain log_10_ PFU/ml.

All graphing and statistical tests were performed in Prism Graphpad (version 8, https://www.graphpad.com/). χ^2^ contingency tests were used to calculate dissemination and transmission rates. Two‐way ANOVA (analysis of variance) with Geisser–Greenhouse correction was used to determine differences in viral growth kinetics. One‐way ANOVA was used to determine differences in bloodmeal titres.

## 3 RESULTS

### 3.1 DDVax variant analysis

The RVFV ZH501 genome deletions used to produce DDVax were reported in Bird et al. (2008), Bird et al. (2007) and Gerrard et al. (2007). We used sequencing to track the genetic stability of DDVax over five passages in Vero cell culture (P1 through P5, MOI 0.0005). Because the resulting vaccine virus is intended for use as a human vaccine virus, we wanted to ensure the absence of DVGs and estimate the rate of coding changes over early passages. The P5 preparation was used for goat inoculations. Total RNA from virus preparations (supernatant: P1–P4, or filtered supernatant: P5) was converted into shotgun Illumina libraries and sequenced on one Illumina NextSeq 500 instrument run to produce a median of 1.2 × 10^7^ single end 150 nucleotide (nt) reads per sample, 88% of which remained after removing low‐quality and adapter‐derived bases. A median of 3.4 × 10^6^ reads (28%) remained after removing reads mapping to the host cell genome (*Chlorocebus sabeus*). A median of 4.4 × 10^5^ reads mapped to the DDVax reference sequence at 6055× coverage across all viral segments. SNVs and short insertion and deletions were quantified using lofreq, and larger structural variants, including possible DVGs, were quantified using DI‐tector (Beauclair et al., 2018; Wilm et al., 2012).

**FIGURE 1:**
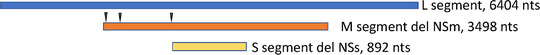
**DDVax schematic**. DDVax was generated from the parental virulent strain ZH501 and contains complete gene deletions of NSm and NSs. Variants were identified in passage 5 (Table 1). Carat indicates SNVs at nt positions 31, 499 and 926 of segment M

Three SNVs in the glycoprotein precursor gene rose to above 50% frequency by P5 (Table 1 and Figure 1). A variant at nucleotide position 31 (relative to the 5′ end of the RVFV M segment complementary RNA) predicted to produce the amino acid change G3E in the DDVax NSm‐deleted glycoprotein (equivalent to Gly 133 in the RVFV NSm/Gn/Gc polyprotein, NC_014396) rose to 54% frequency by P5. A variant at nucleotide position 499 (G159D, equivalent to Gly 289 in ZH501) rose to 55% by P5. And a variant at position 926 (N301K, equivalent to Asn 431 in ZH501) rose to 90% frequency by P5. The highest frequency L segment variant was a synonymous variant at position 4665 that rose to 16% by P5. No variants in the S segment rose above 3% frequency in any sample. Lofreq did not identify any short insertion or deletion variants above 3%. Similarly, DI‐tector did not identify any structural variants (larger insertions, deletions, incomplete transcripts consistent with DVGs or copy‐back variants) with a frequency ≥3%. These variants could have risen in frequency as a result of cell culture adaptation or as a result of genetic drift.

**TABLE 1:** Single nucleotide variants

Segment	Position (nt)^a^	Coding impact	Reference base	Variant base	P1	P2	P3	P4	P5
L	4665	E1549E	A	G	0.00	0.07	0.09	0.12	0.16
L	5483	K1822M	A	T	0.04	0.03	0.03	0.03	0.04
L	5488	D1824Y	G	T	0.07	0.07	0.07	0.07	0.07
L	5513	R1832I	G	T	0.00	0.00	0.00	0.00	0.03
L	6113	Y2032C	A	G	0.05	0.05	0.05	0.04	0.03
M	31^b^	G3E	G	A	0.00	0.00	0.00	0.25	0.54
M	32^b^	G3G	G	A	0.00	0.00	0.07	0.20	0.19
M	190	G56E	G	A	0.00	0.00	0.00	0.00	0.09
M	300	K93E	A	G	0.00	0.00	0.00	0.03	0.05
M	457	Y145C	A	G	0.00	0.00	0.00	0.06	0.00
M	462	L147I	C	A	0.00	0.06	0.09	0.09	0.00
M	499	G159D	G	A	0.00	0.00	0.00	0.11	0.55
M	808	R262K	G	A	0.00	0.07	0.07	0.09	0.00
M	818	K265N	A	T	0.00	0.00	0.00	0.00	0.04
M	925^a^	N301I	A	T	0.00	0.00	0.03	0.03	0.00
M	926^a^	N301K	T	A	0.06	0.34	0.40	0.49	0.90
M	1240	D406G	A	G	0.04	0.00	0.04	0.06	0.00
M	1473	P484S	C	T	0.00	0.00	0.00	0.07	0.00
M	2480	R819R	G	A	0.00	0.00	0.00	0.00	0.05

^a^ Nucleotide position 1 corresponds to the underlined base in the canonical RVFV antigenomic end sequence: 1‐ACACAAAG.

^b^ The variants at positions 31 and 32, and those at positions 925 and 926 are not linked.

#### 3.1.1 Mosquito vector competence

**FIGURE 2:**
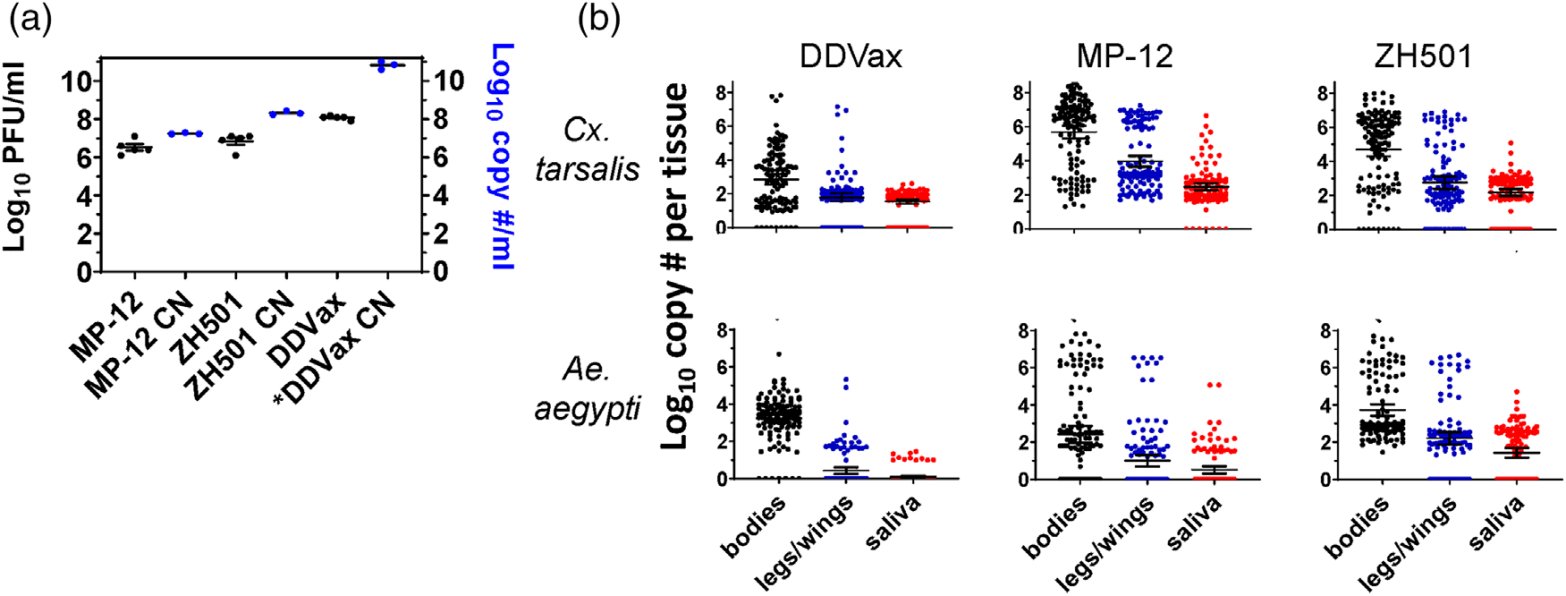
**Bloodmeals and viral RNA detection in RVFV DDVax, MP‐12 and ZH501 in vitro challenged mosquito bodies, legs/wings and salivary expectorants at 14 dpi**. (a) Oral blood meal titres from each of the RVFV strains, DDVax, MP‐12 and ZH501 (left *Y* axis, one‐way ANOVA, *p* = 1.8e‐5). RNA was also extracted from these meals for determination of Log_10_ RNA copy numbers (CN) (right *Y* axis). *DDVax CN: the RNA CN for DDVax was estimated from a similar, but non‐identical bloodmeal used for the dose–response assays described below. RNA extractions represent 3 biological replicates. (b) RVFV RNA detected by RT‐qPCR of bodies, legs/wings and saliva from mosquitoes after virus exposure. Sample positivity rates are listed in Table S1. Viral copy number was calculated using a standard curve of diluted L segment transcripts amplified from a plasmid using in vitro transcription. Profiles from three biological replicates were combined, with approximately 40 mosquitoes per replicate. Horizontal lines indicate mean and 95% confidence intervals. qPCR cut‐off values used a cycle threshold of 40

To measure differences in viral infection kinetics, *Ae. aegypti* and *Cx. tarsalis* were challenged with 1:1 mixtures of blood and freshly grown DDVax and then compared against those infected with MP‐12 or the ZH501 parental strain. Because of the need to use freshly grown virus for infections, it was not possible to control for differences in bloodmeal titres. Mean bloodmeal titres ranged from ∼8.1 logs/ml with DDVax to 6.5 or 6.8 log_10_ PFU/ml in MP‐12 and ZH501, respectively (Figure 2a). Thus, DDVax titres were significantly higher than that of the other two virus strains (ANOVA, *p* = 1.8e‐5). Bloodmeal RVFV RNA copy numbers were also determined (Figure 2a). Log_10_ RNA copy numbers were about 2.75, 0.73 and 1.48 logs higher than Log_10_ PFU/ml of DDVax, MP‐12 and ZH501, respectively. Viral RNA was then detected in *Cx. tarsalis* bodies, legs/wings and saliva at 14 days post‐infection (Figure 2b and Table S2). Saliva samples were also assayed by plaque assay for detection of infectious virus (Table 2).

**TABLE 2:** Proportion of mosquitoes with infectious virus in saliva following RVFV artificial membrane feeding

Species	Virus	# samples	Saliva CPE positive (%)
*Ae. aegypti*	DDVax	120	0 (0%)
	MP‐12	120	19 (16%)
	ZH501	118	32 (27%)
*Cx. tarsalis*	DDVax	120	1 (<1%)
	DDVax	20^a^	0 (0%)
	MP‐12	32	15 (47%)
	ZH501	110	90 (82%)
	ZH501	15^a^	10 (67%)

^a^ No freeze‐thaw.

The percentage of *Culex* mosquito DDVax viral RNA‐positive bodies was not statistically different from MP‐12 or ZH501 infections (Figure 2b and Table S2). However, mean RNA genome copy numbers in *Culex* bodies infected with DDVax were at least two log_10_ values lower than those infected with either MP‐12 or ZH501 strains (Figure 2b, unpaired *t*‐test, *p *= 2.2e‐16, *p *= 4.1e‐09, respectively), though mosquitoes were exposed to a DDVax titre over one log_10_ PFU greater than controls. Dissemination of DDVax viral RNA to *Culex* legs/wings was also significantly reduced compared to MP‐12 (*χ* ^2^ test, *p* = 2.078e‐07). Moreover, infectious DDVax was detected in only 1 of 140 *Culex* saliva samples at 14 dpi, whereas 47% and 82% of MP‐12 and ZH501 infected saliva samples, respectively, showed CPE consistent with the presence of infectious virus (Table 2, *χ* ^2^ test, *p* = 5.3e‐13 vs. MP‐12, 2.8e‐9, vs. ZH501). To rule out the possibility that sample freeze‐thaw compromised virus viability, an additional subset of saliva samples from 14 dpi DDVax exposed mosquitoes were assessed for the presence of infectious virus; still, none was detected (Table 2).

*Ae. aegypti* from the in vitro virus exposure experiments also showed significantly reduced dissemination in DDVax‐infected mosquitoes compared to those challenged with MP‐12 or ZH501, respectively (*χ* ^2^ test, vs. MP‐12 *p* = .02, vs. ZH501 *p* = 2.2e‐16), as indicated by the presence of viral RNA in legs/wings (Figure 2b). *Aedes aegypti* mosquitoes exposed to DDVax had no evidence of infectious virus in expectorated saliva, whereas 16% and 27% of saliva samples were CPE‐positive in MP‐12‐ and ZH501‐infected mosquitoes, respectively (Table 2, *χ* ^2^ test, vs. MP‐12 *p* = 2.2e‐16, vs. ZH501 2.821e‐09).

### 3.2 Dose–response curve

We expected that DDVax would not be found at significant levels outside mosquito midguts, as described in previous reports of plaque assays for infectious virus (Crabtree et al., 2012). Subsequently, our challenge experiments showed unexpectedly high levels of DDVax RNA‐positive, CPE‐negative saliva samples (Tables 2 and S2 and Figure 2b). We hypothesized that the high levels of DDVax viral RNA in saliva may have been due to the high viral titre of the infectious bloodmeal (Figure 2a), which could have overwhelmed natural infection barriers. Therefore, to confirm that viral RNA positivity varied as a function of bloodmeal titre, a second DDVax challenge was performed with *Cx. tarsalis* mosquitoes, using virus serial dilutions. Bloodmeals containing 6.2, 4.5 and 3.5 log_10_ PFU/ml DDVax were tested. There was a trend for reduction of viral RNA in bodies, legs/wings and saliva samples as the bloodmeal titre decreased (Table S2 and Figure S2). However, strikingly, there was still detectable viral RNA in salivary expectorants with all viral dilutions, including the 3.5 log_10_ PFU/ml virus meal.

### 3.3 Mosquito challenge on inoculated goats

**FIGURE 3:**
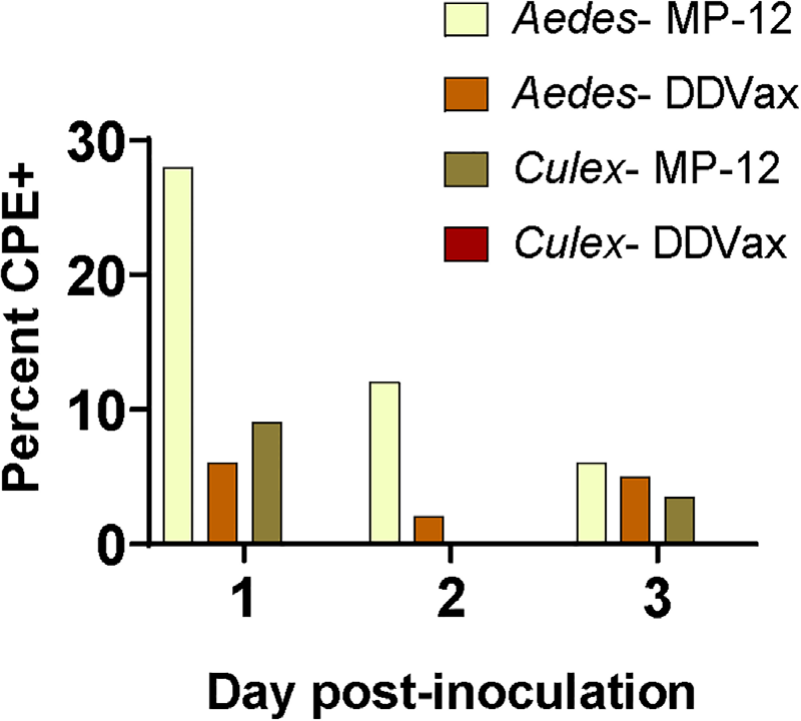
**Infectious DDVax or MP‐12 detected in bodies from mosquitoes fed on inoculated goats**. *Aedes* or *Culex* mosquitoes were fed on goats (*n* = 3 per virus strain) and were held for 7 days prior to determining infectious load by plaque assay (Table 3). Graph shows percentage of bodies at each day post‐inoculation that were CPE positive, indicative of infectious virus. *Aedes* DDVax, *n* = 50, 50 and 98 for days 1, 2 and 3, respectively. *Aedes* MP‐12, *n* = 64, 60 and 100 for days 1, 2 and 3, respectively. *Culex* DDVax, *n* = 11, 8 and 40 for days 1, 2 and 3, respectively, showed no evidence of infectious virus at any timepoint. *Culex* MP‐12, *n* = 22, 9 and 56 for days 1, 2 and 3, respectively

To further test the environmental safety profile of DDVax, goats were inoculated with either DDVax or MP‐12 viruses. Mosquitoes were allowed to directly feed on the goats at 1 and 2 days post‐inoculation (Figure S1). On day 3, blood was collected into EDTA tubes and transferred to water‐jacketed feeders for mosquito challenge in the laboratory. Numbers of engorged mosquitoes from each daily goat feeding are listed in Table 3. Sera from all goat blood specimens were negative for DDVax or MP‐12 by plaque assay at 1, 2 and 3 dpi (limit of detection 1 log_10_ PFU/ml). However, trace levels of viral RNA were detectable by RT‐qPCR (Figure S3). After a 7‐day extrinsic incubation period, *Aedes* and *Culex* bodies showed evidence of infectious MP‐12 by plaque assay (Figure 3), indicative of midgut infections, as previously described (Crabtree et al., 2012; Kading, Crabtree, et al., 2014). Viral prevalence was highest in *Aedes* (28%) exposed to goats at 1 day post‐vaccination with MP‐12 strain; these *Aedes* mosquito infection rates decreased to 12% and 6% in mosquitoes that fed on goats 2 and 3 days post‐vaccination, respectively. In contrast, 6% (day 1), 2% (day 2) and 5% (day 3) of *Aedes* mosquitoes that fed on DDVax‐inoculated goats were positive for infectious virus by CPE assay after a 7‐day incubation period. Across the time series, *Aedes* mosquitoes exposed to MP‐12 vaccinated goats showed significantly higher rates of virus‐positive bodies than those exposed goats inoculated with DDVax (*χ* ^2^ test, *p *= .011). *Culex* showed low rates of MP‐12 virus infection (≤ 10%) and no evidence of infection with DDVax. Specifically, 4 of 87 *Culex* mosquitoes that fed on goats vaccinated with MP‐12, and 0/59 *Culex* mosquitoes that fed on goats inoculated with DDVax, showed evidence of infection after a 7‐day incubation. The differences in *Culex* were not significant (Table 3). All mosquito bodies that were CPE‐positive were assessed for the presence of disseminated live virus in legs/wings. However, none of the mosquitoes that became infected after feeding on inoculated goats showed evidence of infectious virus in disseminated infection (positive legs/wings).

**TABLE 3:** Viral CPE in bodies at 7 days post‐goat exposure

	DAY1		DAY2		DAY3^a^		Totals	
Virus	***Culex***	***Aedes***	***Culex***	***Aedes***	***Culex***	***Aedes***	***Culex***	***Aedes***
**MP‐12**	2/22 (10%)	18/64 (28%)	0/9 (0%)	7/60 (12%)	2/56 (4%)	6/100 (6%)	**4/87 (4.6%)**	**31/224 (13.8%)**
**DDVAX**	0/11 (0%)	3/50 (6%)	0/8 (0%)	1/50 (2%)	0/40 (0%)	5/98 (5%)	**0/59 (0.0%)**	**9/198 (4.6%)**

^a^ Goat blood was collected into EDTA tubes and then provided to mosquitoes through an artificial feeder.

### 3.4 Viral growth curves in mosquito cell lines

To further characterize DDVax replication kinetics compared to MP‐12 and ZH501 strains, growth curves were performed in three insect cell lines. Aag2 (*Ae. aegypti*, embryonic), ATC‐10 (*Ae. aegypti*, larval) and Ct (*Cx. tarsalis*, embryonic) cells were infected with DDVax, MP‐12 or ZH501 over 6‐day time courses. The *Aedes aegypti* larval cell line ATC‐10 was not susceptible to infection with any virus strain. DDVax replicated in Aag2 cells to lower peak titres than did MP‐12 or ZH501 strains (Figure S4) (random effects mixed model ANOVA, *p* = 8.0e‐4). Similarly, DDVax also attained lower titres than control viruses in Ct cells (random effects mixed model ANOVA, *p* = 3.5e‐4). MP‐12 grew to similar peak titres in Ct and Aag2 cells, at 9.1 and 9.5 log_10_ PFU/ml, respectively. Peak ZH501 titres were 8.0 and 6.9 log_10_ PFU/ml, in Ct and Aag2 cells, respectively. The virulent strain caused syncytial formation and lifting of cell monolayers, consistent with pathogenicity (Turell et al., 1984), which could have affected final titres. Lastly, mean peak DDVax titres were 7.1 and 6.3 log_10_ PFU/ml, in Ct and Aag2 cells, respectively, which are lower than peak titres for MP‐12 and ZH501. DDVax grew better in Ct cells than in Aag2 cells (two‐way ANOVA, *p *= 4.5e‐5), consistent with the mosquito data.

## 4 DISCUSSION

This study utilized multiple approaches to demonstrate the relative safety of the DDVax vaccine candidate in the context of mosquito transmissibility. Risk of reassortment and reversion to virulence are also of concern. Though these aspects were not addressed here, they are currently under investigation. The current work was designed as part of a series of safety studies in advance of human clinical trials. DDVax showed favourable environmental safety profiles (e.g. low mosquito dissemination and impaired transmission from inoculated livestock) compared to MP‐12 vaccine and the wild‐type parental virus, ZH501. In artificial feeding experiments, mosquitoes from two epidemiologically relevant genera were challenged with viral titres up to 2–5 log_10_ PFU/ml higher than mosquitoes would be expected to encounter in the field from vaccinated animals, and there was only one questionably positive transmission event. In a previous study, sheep vaccinated with DDVax did not develop any detectable vaccine‐associated viremia following inoculation, suggesting that the overall burden of DDVax in animals is very low (Bird et al., 2011). Additionally, DDVax viral RNA copy numbers in bodies and legs/wings were significantly reduced in both *Aedes* and *Culex* compared to those infected with either MP‐12 or ZH501 (Figure 2b). This result is consistent with the previously observed impaired viral dissemination phenotype in mosquitoes due to the deletion of the NSm coding region (Crabtree et al., 2012; Kading, Crabtree, et al., 2014). Deletion of NSm alone, or NSm and NSs, significantly inhibited mosquito infection and transmission potential as compared with deletion of NSs alone (Crabtree et al., 2012). Only 1 of 140 mosquito saliva samples contained live DDVax virus (Table 2), which was also consistent with previous experiments (Crabtree et al., 2012). This single positive saliva sample showed a single plaque, which may not have been infectious and for which we cannot rule out the possibility that it represented low‐level contamination. Expected virus infection rates in these mosquito species have previously ranged between 63% and 84% for virulent RVFV in *Ae. aegypti* (Crabtree et al., 2012; Kading, Crabtree, et al., 2014) and 58% and 72% for *Cx. tarsalis* (Bergren et al., 2021; Turell et al., 2010). Forty‐one per cent of *Cx. pipiens* mosquitoes became infected with MP‐12 strain following artificial challenge in a blood meal containing 10^4.1^ pfu/ml (Turell & Rossi, 1991). In contrast, we expected 0% DDVax infection in *Ae. aegypti* (Crabtree et al., 2012). Overall, in this work, we observed similar results between ZH501 and MP‐12 strains, with a significant reduction in infection of mosquitoes with DDVax.

While DDVax RNA was detectable in multiple body compartments of the mosquito, infectivity was very reduced given the low RNA copy number detected in mosquitoes 14 days post in vitro infection (Figure 2b). For example, if mosquitoes imbibed a 5 μl blood meal of 10.8 log_10_ copies/ml, then 8.5 log_10_ DDVax copies would have been acquired. In our study, after 2 weeks incubation, 2.9 log_10_ mean RNA copies were detected in *Culex* bodies, 1.8 log_10_ RNA copies in legs/wings and 1.5 log_10_ RNA copies in saliva, suggesting that the virus may have somehow disseminated and persisted at a low level, but was not actively replicating. By comparison, mosquitoes of each species exposed to MP‐12 and ZH501 had RVFV RNA copy numbers between 7 and 8 log_10_ by 14 days post‐exposure (Figure 2b) after exposure to a blood meal containing over an order of magnitude less virus than that of DDVax (Figure 2a). This pattern was consistent with the results of the dose–response experiment, in which the RNA copy number in different tissue compartments appeared to be relatively stable after 14 days across all three exposure doses (Figure S2). It is not clear how retention of viral RNA occurred. Further, our data showed that RNA copy numbers exceeded infectious titres, rendering the infectious virus population even lower (Wichgers Schreur et al., 2021).

Consistent with these findings, Kading, Crabtree, et al. (2014) reported 80% infection and 60% dissemination rates of rZH501 (recombinant ZH501) by *Ae. aegypti* mosquitoes, compared with 0% infection and 0% dissemination rates of the rZH501‐delNSm (NSm deletion) strain, by plaque assay. Nevertheless, in rZH501‐delNSm infections, viral protein was detected in most mosquitoes by immunofluorescence assay (IFA), consistent with successful viral protein translation in the presence of defective packaging or cellular egress. Moreover, IFA foci in the midguts of mosquitoes infected with rZH501‐delNSm were also very small compared with extensive midgut foci characteristic of rZH501 (Kading, Crabtree, et al., 2014). Therefore, detection of DDVax RNA and whether viral proteins are detected outside the midgut, in the absence of infectious virus, warrants further study.

The presence of DDVax RNA in *Culex* saliva could be the result of cell‐to‐cell spread of the virus through tissues in the absence of efficient viral assembly, or possibly ‘leakage’ of virions from the alimentary tract in the absence of viral replication. Romoser and colleagues reported the particular affinity of virulent ZH501 RVFV for the cardia, intussuscepted foregut, fat body and salivary glands in *Culex pipiens* mosquitoes (Lerdthusnee et al., 1995; Romoser et al., 1992). The cardia and intussuscepted foregut are transitional tissues between the oesophagus and the anterior midgut in the mosquito digestive tract (Romoser et al., 1992). Salivary glands are proximal to this region, embedded in the fat body. One possible explanation is that DDVax retained similar tissue affinity in the absence of NSs and NSm, and, when combined with presumed less efficient viral assembly, led to detection of viral RNA but no infectious virus (Figure 2b and Tables 2 and S1). In addition, Romoser et al. (1992) reported that, in *Culex*, RVFV ZH501 was able to escape to peripheral tissues as early as 1 day following an infectious blood meal, making it particularly rapid in its dissemination compared to other arboviruses, for example, flaviviruses, which often require at least a week to reach the salivary glands (Sanchez‐Vargas et al., 2009), depending on extrinsic incubation temperature. RVFV affinity for salivary glands was substantiated by the DDVax dose–response experiment, in which nearly 19% of mosquitoes showed viral RNA in salivary expectorants at the lowest bloodmeal titre of 3.5 log_10_ PFU/ml (Table S3).

To address concern about the presence of one DDVax PFU in a single saliva sample, *Cx. tarsalis* mosquitoes were subsequently challenged with artificial blood meals containing a range of viral titres. As expected, the percentage of mosquitoes that became infected, as determined by RNA genome copy number, decreased proportionally with the titre of DDVax in the artificial blood meal, but did not reach zero. The stable persistence of DDVax RNA in different tissue compartments was evident in all dosing groups (Figure S2). As experimentally predicted, the higher the blood meal titre, the higher the percentage of mosquitoes had detectable RNA, although infectious virus was not assayed in mosquitoes challenged with lower titre blood meals.

These results were further confirmed and placed into a realistic epidemiological context by feeding mosquitoes on inoculated goats. Infection of goats with wild‐type ZH501 was not possible in this study due to biosafety considerations. Mosquitoes were fed on goats on days 1–3 post‐inoculation with DDVax or MP‐12. As expected, goats did not develop any detectable viremia, as determined by plaque assay. However, small ruminants, for example, sheep, would be expected to develop a viremia ranging from ∼5 to 6 log_10_ TCID_50_/ml titres between 1 and 3 days post‐infection with a wild‐type strain (Wichgers Schreur et al., 2021). Similarly, neither Wilson et al. (2014) nor Nyundo et al. (2019) observed any detectable viremia in ruminants following vaccination with MP‐12 strain. Morrill et al. (1991) noted a transient, low‐titre viremia in lambs vaccinated with MP‐12 strain. Sheep inoculated with DDVax failed to develop any detectable viremia (Bird et al., 2011). In a very similar study, Miller et al. (2015) fed multiple species of mosquitoes including *Cx. tarsalis* and *Ae. aegypti* on sheep vaccinated with MP‐12 and held mosquitoes for 10–14 days after feeding. No RVFV RNA was detected in any mosquitoes by RT‐PCR (Miller et al., 2015). Therefore, it was surprising to observe that, in this study, mosquitoes fed on these inoculated goats and held for 7 days post‐feeding developed infections (Figure 3 and Table 3).

Analysis of goat serum samples showed very low (<10 RNA copies/ml) RNA levels of RVFV in goat serum (Figure S3), which we interpreted to represent residual, circulating virus as opposed to actively replicating virus. Mosquitoes were able to pick up this residual viral inoculum; however none of these mosquitoes developed a disseminated infection by 7 days post‐exposure. For infection with ZH501, dissemination was previously documented to occur as early as 3 days post‐exposure (Romoser et al., 1992), with all mosquitoes having developed a disseminated infection by 10 days post‐exposure (Kading, Crabtree, et al., 2014).

Mosquito infectivity also becomes a function of volumetric constraints of mosquito blood meal size. While the probability of one mosquito imbibing infectious virions is lower at low virus titres, many mosquitoes imbibing a blood meal simultaneously would draw a larger collective volume of blood that could result in one or more mosquitoes picking up infectious virions. For example, detection of virus in a single mosquito blood meal is limited to titres > 3 log_10_ PFU/ml serum (approximately 1 PFU in 1 μl of serum in a blood meal) (Kading, Crabtree, et al., 2014). For a 25% probability of detecting virus in a single 2 μl mosquito blood meal, the serum titre needs to be 2.72 log_10_ PFU/ml (95% CI 2.19−3.27), while for a 50% probability of detection, the titre needs to be 3.64 log_10_ PFU/ml (95% CI 3.20−4.08) (Kading, Crabtree, et al., 2014). Corresponding titres for 75% and 90% probabilities of detection were 4.56 log_10_ PFU/ml (95% CI 4.02−5.10) and 5.48 log_10_ PFU/ml (95%CI 4.71−6.24), respectively (Kading, Crabtree, et al., 2014).

Wichgers Schreur et al. (2021) documented the extraordinary efficiency of RVFV transmission between lambs and *Ae. aegypti* mosquitoes when using an animal model as opposed to an artificial system. Approximately 30% more RVFV saliva‐positive mosquitoes resulted from feeding on viremic lambs than from feeding on a membrane system (Wichgers Schreur et al., 2021) testifying to the value of conducting these experiments with an in vivo model system to more realistically represent vertebrate infectiousness to mosquitoes. While dissemination of DDVax after our 7‐day timepoint cannot be ruled out, our collective results suggest that transmission risk would be very low because any disseminated virions would not be infectious. In addition, based on previous reports, there was a low combined probability for a single mosquito to imbibe infectious virus (Crabtree et al., 2012; Kading, Biggerstaff, et al., 2014; Kading, Crabtree, et al., 2014), as well as impaired dissemination due to the deletion of the NSm gene (Kading, Crabtree, et al., 2014). Finally, we saw the lack of infectious DDVax expectorated in mosquito saliva even after a high titre virus challenge. These features provide support for a favourable DDVax environmental profile.

## 5 CONCLUSION

Due to the double gene deletion of NSs and NSm, DDVax has less efficient viral replication in mosquitoes than the vaccine strain MP‐12 or wild‐type ZH501. Mosquitoes were able to imbibe and harbour infectious DDVax following a high titre challenge in the lab or by feeding on inoculated goats. However, DDVax replication and dissemination was impaired in mosquitoes, and only one individual mosquito had one DDVax plaque in its saliva after a high titre challenge. Given the combined probability of a single mosquito imbibing an infectious virion precisely after inoculation, the extremely low imbibed virus titre, the impaired dissemination in mosquitoes due to the deletion of the NSm gene and the lack of infectious DDVax expectorated in mosquito saliva even after a high titre virus challenge, the transmission and dissemination of DDVax by mosquitoes from vaccinated individuals in an epidemiologically relevant scenario is highly unlikely.

## ETHICS STATEMENT

The authors confirm that the ethical policies of the journal, as noted on the journal's author guidelines page, have been adhered to and the appropriate ethical review committee approval has been received. The US National Institutes of Health guidelines for the Care and Use of Laboratory Animals were followed.

## CONFLICT OF INTEREST

The authors declare they have no conflict of interest.

## AUTHOR CONTRIBUTIONS

Conceptualization: R.C. Kading, B. Bird, R. Bowen. Formal analysis: C.L. Campbell, M.D. Stenglein. Funding acquisition: R.C. Kading, J. Wyckoff, B. Bird, R. Bowen. Investigation: C.L. Campbell, T.S. Snell, S. Bennett, E.H. Harris, D.H. Hartman, R. Bowen, E. Lian, M.D. Stenglein. Methodology: C.L. Campbell, S. Bennett, D. Heaslip. Validation: C.L. Campbell, D. Heaslip, S. Bennett. Visualization: C.L. Campbell, T.S. Snell, S. Bennett, E. Lian. Writing: C.L. Campbell and R.C. Kading. Resources: J. Wyckoff, D. Heaslip, B. Bird.

## Supporting information

Supporting Information

## Data Availability

The data that support the findings of this study are available from the corresponding author upon reasonable request.
