# Supplementary material for: Safety study of Rift Valley Fever human vaccine candidate (DDVax) in mosquitoes
Source: Transbound Emerg Dis. 2022 Jan 5;69(5):2621–33. doi: 10.1111/tbed.14415 (PMC9788258; doi:10.1111/tbed.14415)
Supplement: Supplementary file 1 — Supporting Information [file TBED-69-2621-s001.pptx]

## Slide 1
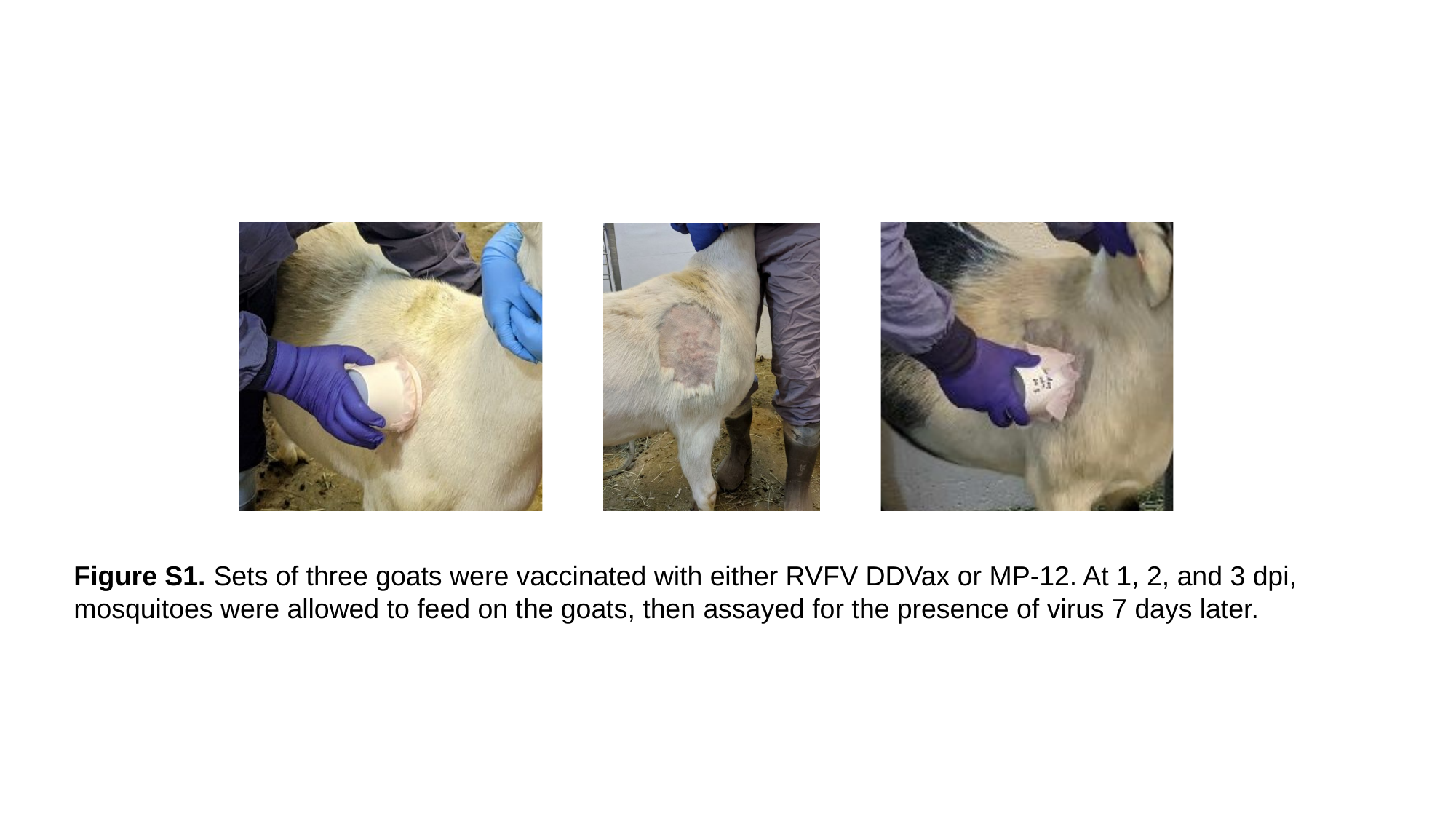

Figure S1. Sets of three goats were vaccinated with either RVFV DDVax or MP-12. At 1, 2, and 3 dpi, mosquitoes were allowed to feed on the goats, then assayed for the presence of virus 7 days later.

## Slide 2
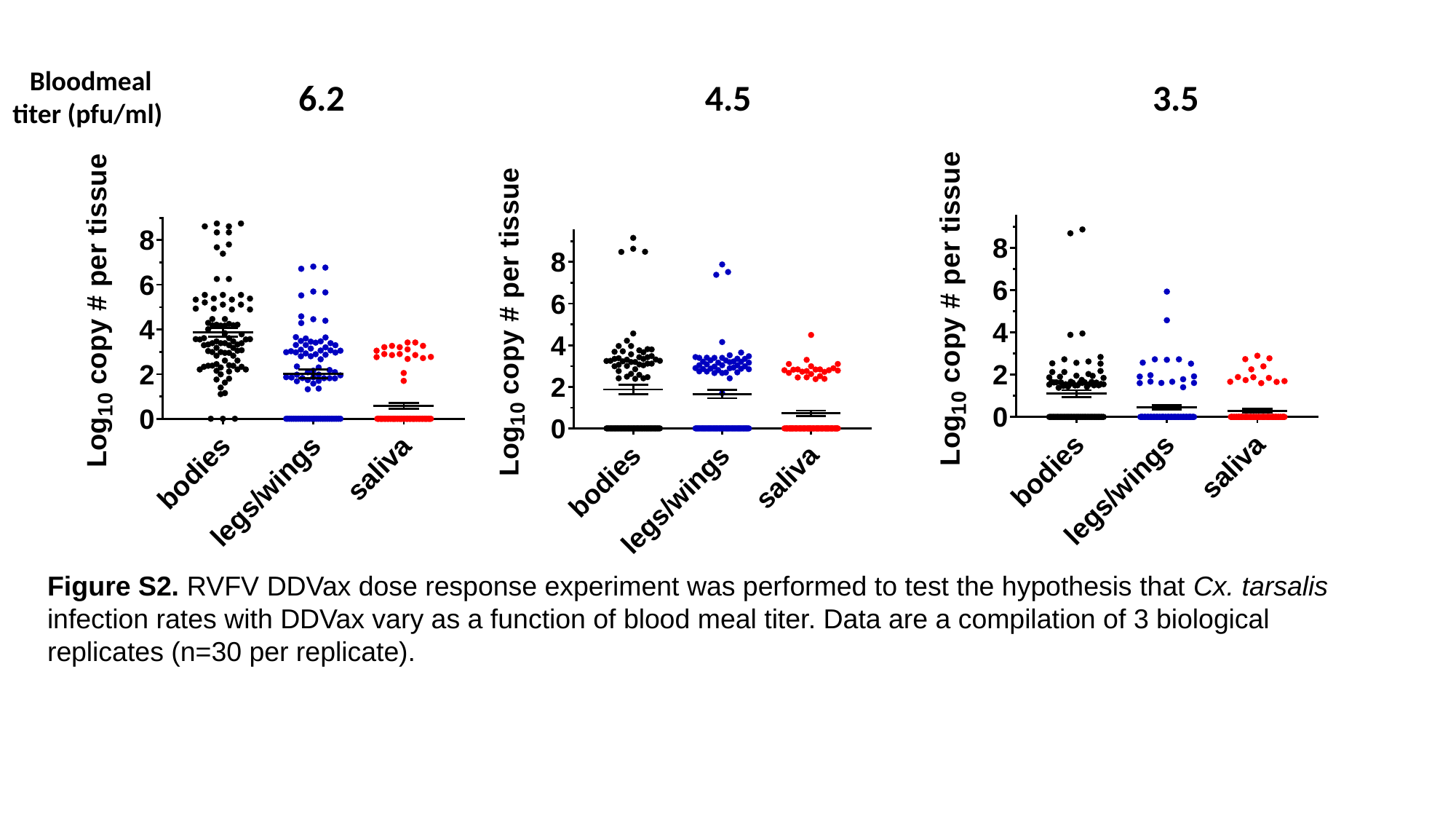

Bloodmeal titer (pfu/ml)
6.2 4.5 3.5
Figure S2. RVFV DDVax dose response experiment was performed to test the hypothesis that Cx. tarsalis infection rates with DDVax vary as a function of blood meal titer. Data are a compilation of 3 biological replicates (n=30 per replicate).

## Slide 3
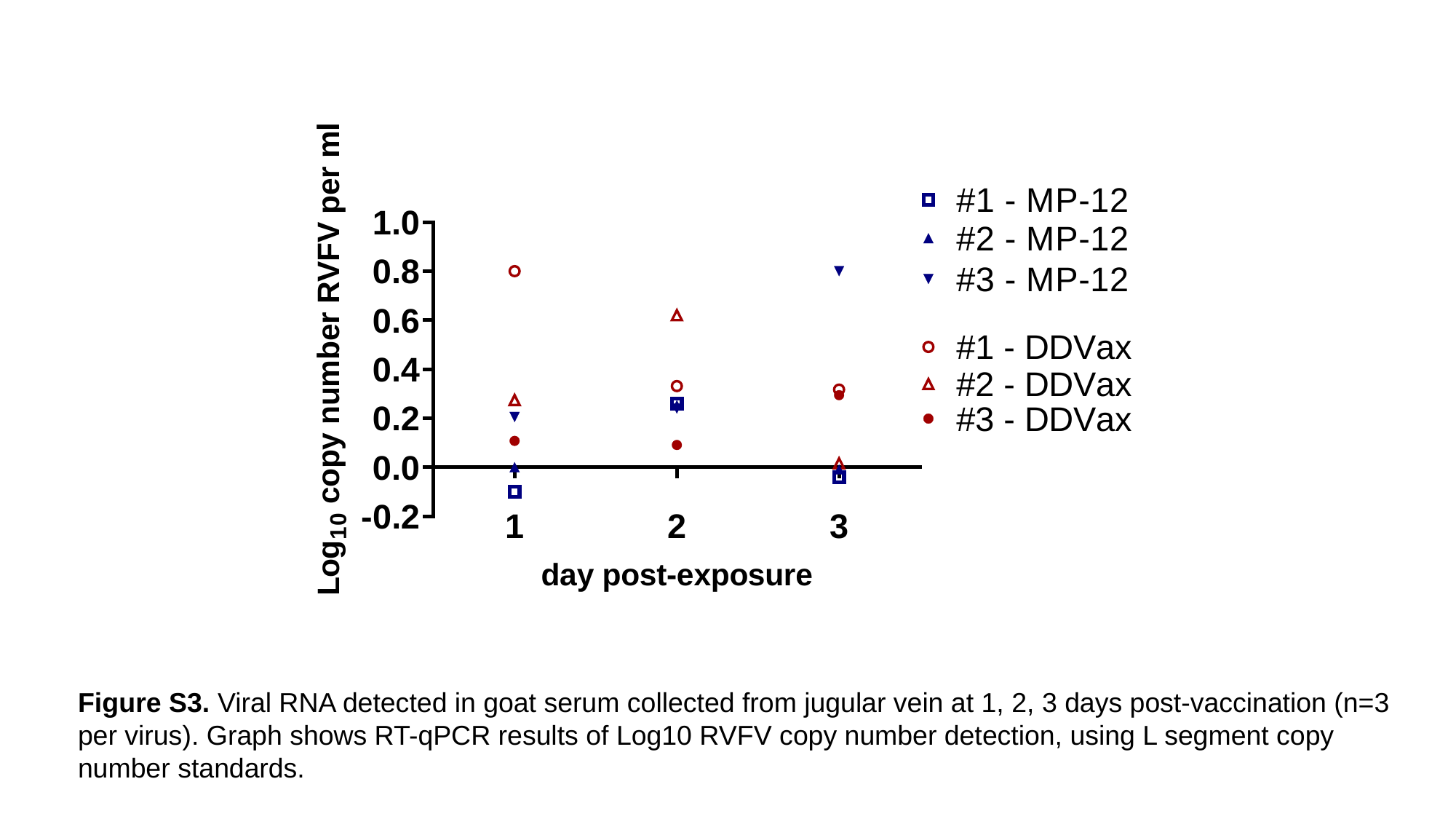

Figure S3. Viral RNA detected in goat serum collected from jugular vein at 1, 2, 3 days post-vaccination (n=3 per virus). Graph shows RT-qPCR results of Log10 RVFV copy number detection, using L segment copy number standards.

## Slide 4
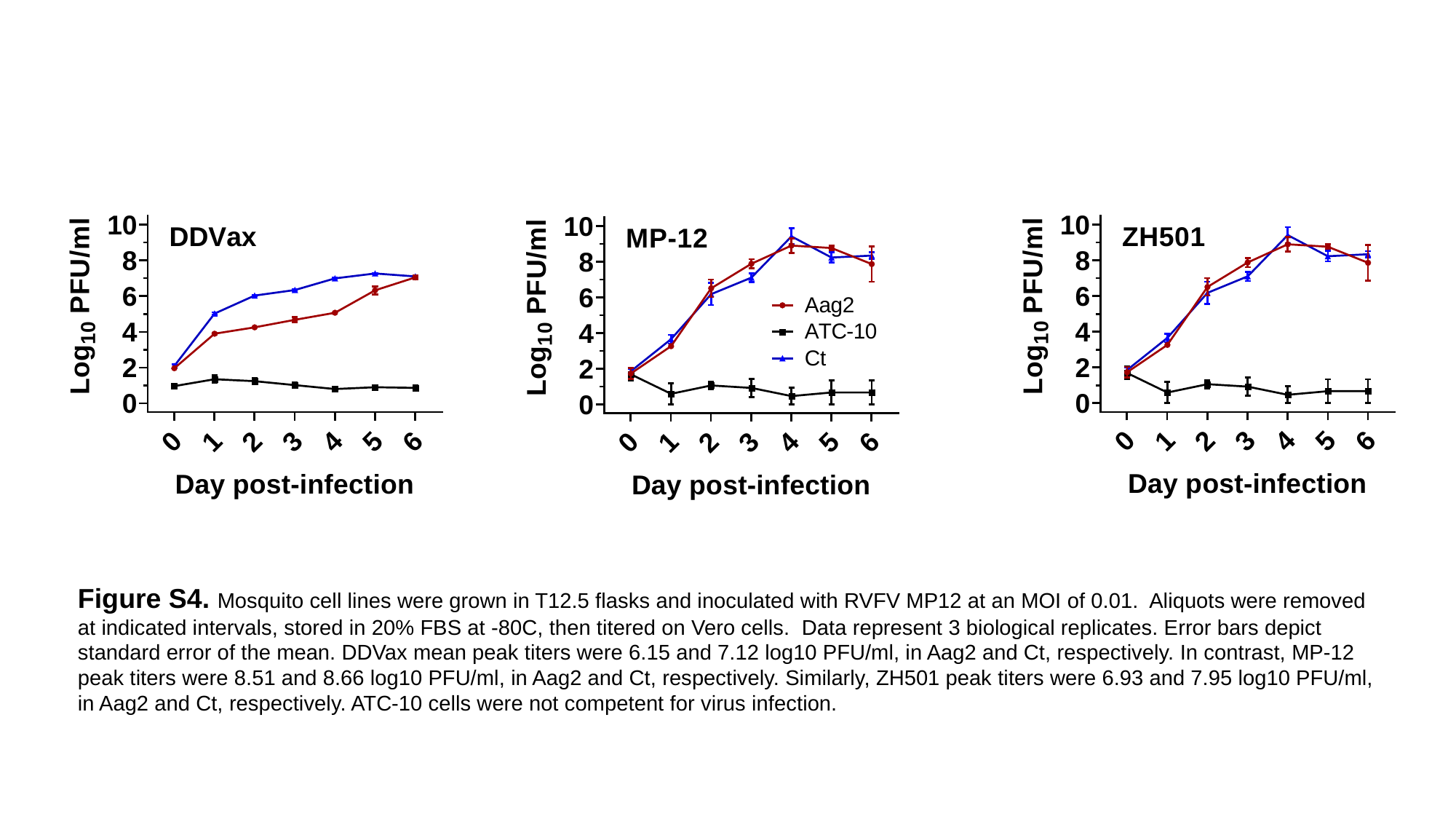

Figure S4. Mosquito cell lines were grown in T12.5 flasks and inoculated with RVFV MP12 at an MOI of 0.01. Aliquots were removed at indicated intervals, stored in 20% FBS at -80C, then titered on Vero cells. Data represent 3 biological replicates. Error bars depict standard error of the mean. DDVax mean peak titers were 6.15 and 7.12 log10 PFU/ml, in Aag2 and Ct, respectively. In contrast, MP-12 peak titers were 8.51 and 8.66 log10 PFU/ml, in Aag2 and Ct, respectively. Similarly, ZH501 peak titers were 6.93 and 7.95 log10 PFU/ml, in Aag2 and Ct, respectively. ATC-10 cells were not competent for virus infection.

## Slide 5
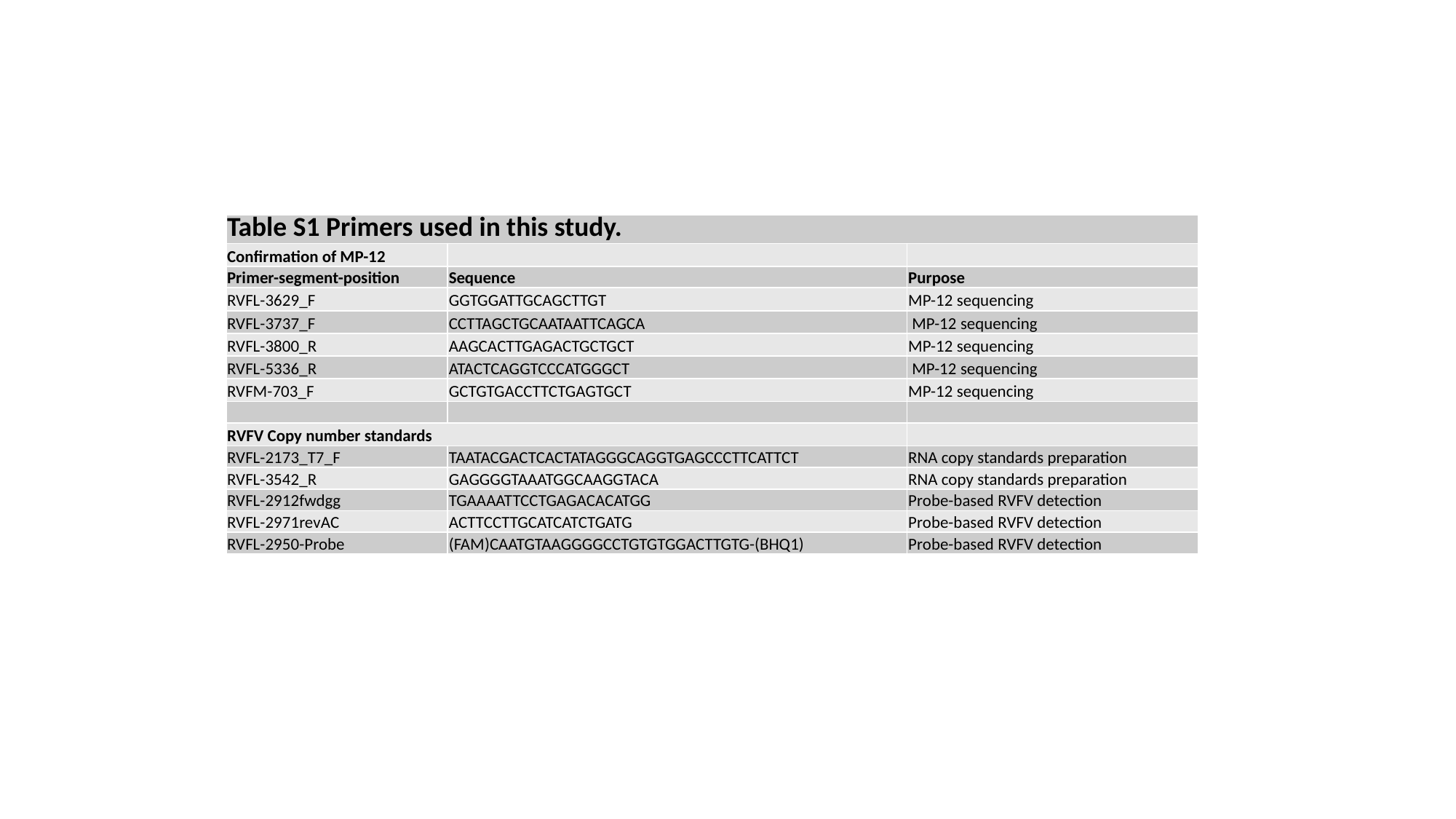

| Table S1 Primers used in this study. | | |
| --- | --- | --- |
| Confirmation of MP-12 | | |
| Primer-segment-position | Sequence | Purpose |
| RVFL-3629\_F | GGTGGATTGCAGCTTGT | MP-12 sequencing |
| RVFL-3737\_F | CCTTAGCTGCAATAATTCAGCA | MP-12 sequencing |
| RVFL-3800\_R | AAGCACTTGAGACTGCTGCT | MP-12 sequencing |
| RVFL-5336\_R | ATACTCAGGTCCCATGGGCT | MP-12 sequencing |
| RVFM-703\_F | GCTGTGACCTTCTGAGTGCT | MP-12 sequencing |
| | | |
| RVFV Copy number standards | | |
| RVFL-2173\_T7\_F | TAATACGACTCACTATAGGGCAGGTGAGCCCTTCATTCT | RNA copy standards preparation |
| RVFL-3542\_R | GAGGGGTAAATGGCAAGGTACA | RNA copy standards preparation |
| RVFL-2912fwdgg | TGAAAATTCCTGAGACACATGG | Probe-based RVFV detection |
| RVFL-2971revAC | ACTTCCTTGCATCATCTGATG | Probe-based RVFV detection |
| RVFL-2950-Probe | (FAM)CAATGTAAGGGGCCTGTGTGGACTTGTG-(BHQ1) | Probe-based RVFV detection |

## Slide 6
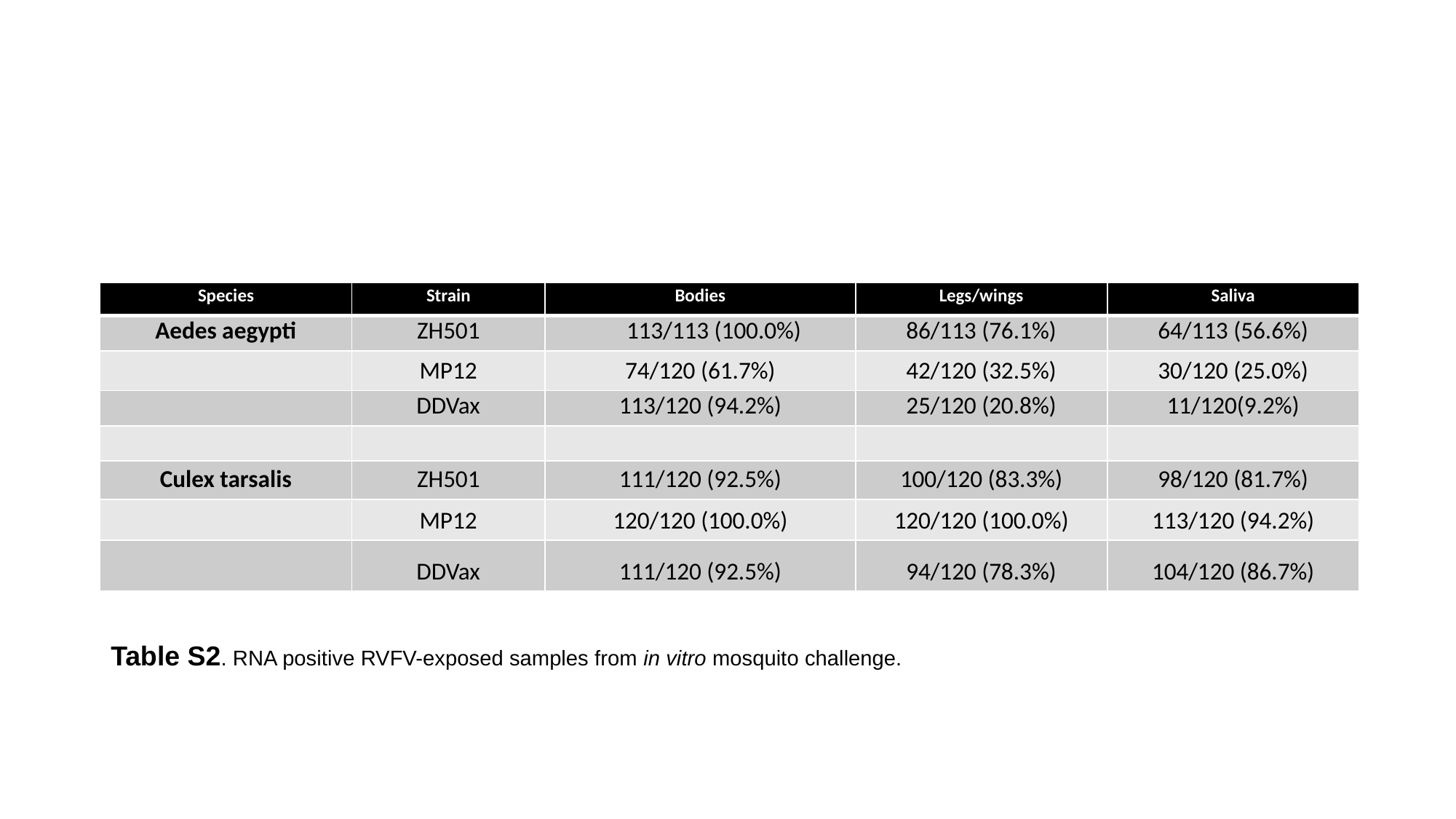

| Species | Strain | Bodies | Legs/wings | Saliva |
| --- | --- | --- | --- | --- |
| Aedes aegypti | ZH501 | 113/113 (100.0%) | 86/113 (76.1%) | 64/113 (56.6%) |
| | MP12 | 74/120 (61.7%) | 42/120 (32.5%) | 30/120 (25.0%) |
| | DDVax | 113/120 (94.2%) | 25/120 (20.8%) | 11/120(9.2%) |
| | | | | |
| Culex tarsalis | ZH501 | 111/120 (92.5%) | 100/120 (83.3%) | 98/120 (81.7%) |
| | MP12 | 120/120 (100.0%) | 120/120 (100.0%) | 113/120 (94.2%) |
| | DDVax | 111/120 (92.5%) | 94/120 (78.3%) | 104/120 (86.7%) |
Table S2. RNA positive RVFV-exposed samples from in vitro mosquito challenge.

## Slide 7
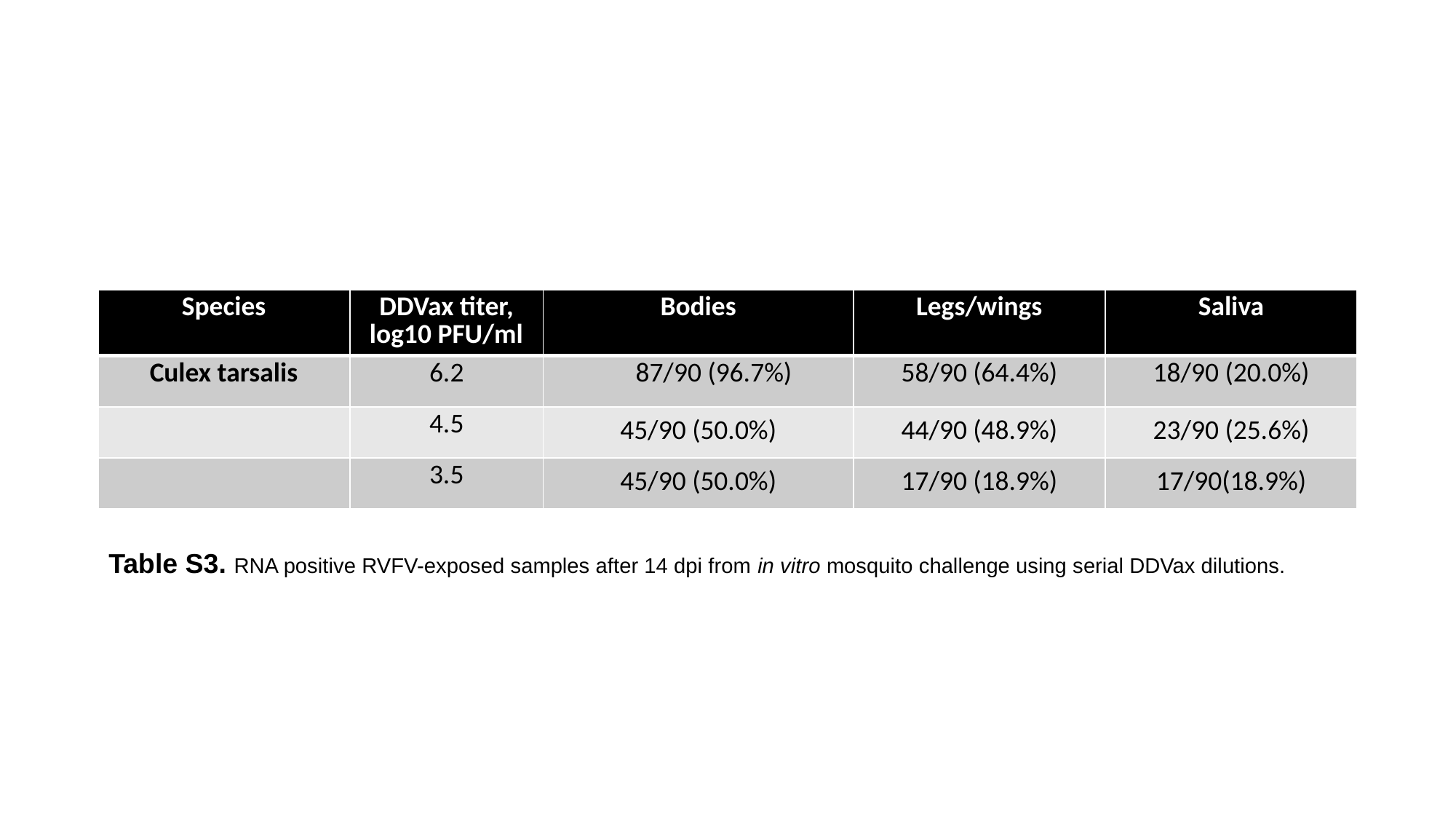

| Species | DDVax titer, log10 PFU/ml | Bodies | Legs/wings | Saliva |
| --- | --- | --- | --- | --- |
| Culex tarsalis | 6.2 | 87/90 (96.7%) | 58/90 (64.4%) | 18/90 (20.0%) |
| | 4.5 | 45/90 (50.0%) | 44/90 (48.9%) | 23/90 (25.6%) |
| | 3.5 | 45/90 (50.0%) | 17/90 (18.9%) | 17/90(18.9%) |
Table S3. RNA positive RVFV-exposed samples after 14 dpi from in vitro mosquito challenge using serial DDVax dilutions.
